# Is the MRI Phenotype Associated with Survival in Patients with Different Breast Cancer Subtypes?

**DOI:** 10.3390/cancers18203309

**Published:** 2026-10-14

**Authors:** Carla Chizuru Tajima, Soraia Quaranta Damião, Cynthia Aparecida Bueno de Toledo Osorio, Marina Sonagli, Solange Moraes Sanches, Almir Galvão Vieira Bitencourt

**Affiliations:** 1 Department of Imaging, Beneficência Portuguesa de São Paulo, São Paulo 01323-001, Brazil; carla.tajima@accamargo.org.br; 2 Graduate Program, A.C. Camargo Cancer Center, São Paulo 01509-010, Brazil; 3 Department of Imaging, A.C. Camargo Cancer Center, São Paulo 01509-010, Brazil; soraia.damiao@accamargo.org.br; 4 Department of Pathology, A.C. Camargo Cancer Center, São Paulo 01509-010, Brazil; cynthia.osorio@accamargo.org.br; 5 Department of Breast Surgery, A.C. Camargo Cancer Center, São Paulo 01509-010, Brazil; marina.sonagli@accamargo.org.br; 6 Department of Clinical Oncology, A.C. Camargo Cancer Center, São Paulo 01509-010, Brazil; solange.sanches@accamargo.org.br

**Keywords:** breast neoplasms, magnetic resonance imaging, triple-negative breast neoplasms, prognosis, disease-free survival, phenotype

## Abstract

Background: Breast cancer exhibits marked molecular and phenotypic heterogeneity, with important implications for prognosis and therapeutic response. Although magnetic resonance imaging (MRI) is widely used for detection and staging, its potential role as a prognostic biomarker across molecular subtypes remains incompletely understood. This study aimed to evaluate the association between MRI-defined tumor imaging phenotypes and disease-free survival (DFS) among different breast cancer molecular subtypes. Methods: This retrospective single-center study included 407 patients with invasive breast carcinoma of no special type who underwent pre-treatment breast MRI between October 2015 and May 2021. Tumors were classified into molecular subtypes and three MRI phenotypes based on lesion morphology and distribution. Associations between imaging phenotype, molecular subtype, and DFS were assessed using Kaplan–Meier analysis and Cox proportional hazards models. Results: Type 3 was the most prevalent imaging phenotype overall (60%), followed by Type 2 (21.9%) and Type 1 (18.2%). Imaging phenotype distribution differed significantly among molecular subtypes. Over a median follow-up of 57 months, imaging phenotype was not associated with DFS in Luminal A, Luminal B, or HER2-overexpressed tumors. However, in the triple-negative/ER-low subgroup, imaging phenotype demonstrated an independent association with DFS. In multivariable analysis, patients with the Type 1 phenotype had an 8.16-fold higher risk of recurrence compared with those with the Type 2 phenotype (95% CI: 1.49–44.60; *p* = 0.015). Type 2 lesions showed the most favorable survival outcomes. Conclusions: In this exploratory analysis, MRI phenotype was associated with disease-free survival in triple-negative and ER-low breast cancers. Circumscribed, unifocal tumors are associated with improved survival, whereas spiculated or multifocal patterns are associated with worse outcomes. However, given the small number of events in this subgroup, these exploratory findings need to be confirmed in larger studies before MRI morphology can be considered a prognostic imaging biomarker in aggressive breast cancer subtypes.

## Simple Summary

Breast cancer is not a single disease but a group of tumors that behave very differently from one another. In this study of 407 women, we examined whether tumor appearance on breast magnetic resonance imaging is associated with long-term outcomes. In an exploratory analysis, MRI phenotype was associated with disease-free survival in triple-negative and ER-low breast cancer. Circumscribed, unifocal tumors (Type 2 phenotype) are associated with significantly better disease-free survival, whereas spiculated or multifocal patterns (Types 1 and 3) are associated with worse outcomes in these aggressive subtypes. MRI morphology may provide prognostic information beyond molecular subtype in triple-negative breast cancer; these exploratory findings require validation in larger prospective cohorts.

## 1. Introduction

Breast cancer is a heterogeneous and complex disease, whose molecular subtypes present different risk factors, treatments, and prognoses [1,2]. Based on gene expression profiles, tumors can be classified into four main subtypes: Luminal A, Luminal B, HER2 overexpressed, and triple negative (TN) [3]. In general, luminal tumors have a more favorable prognosis due to hormone receptor positivity, with the Luminal A subtype being associated with the best survival outcomes. The Luminal B subtype accounts for approximately 40% of cases and, although more aggressive than Luminal A, still demonstrates a better prognosis than non-luminal subtypes [4,5]. TN tumors, which represent about 10–15% of cases in Western countries, tend to affect younger patients and are associated with more-severe clinical outcomes [6]. Luminal tumors with estrogen receptor (ER) expression between 1% and 10% are classified as ER-low positive and exhibit biological characteristics more like TN tumors [7,8,9].

Breast magnetic resonance imaging (MRI) is the most sensitive imaging modality for breast cancer detection, allowing detailed characterization of lesions by morphology, distribution, and dynamic contrast enhancement patterns [10,11]. MRI is particularly useful in patients with multifocal or multicentric disease or aggressive subtypes, such as triple-negative and HER2-overexpressed tumors [12,13]. Studies have shown that MRI findings may reflect the histopathological and molecular characteristics of the tumor [14,15]. Thus, MRI not only aids in detection and preoperative staging but also provides insights into tumor biological behavior, contributing to risk stratification and personalized therapeutic planning.

Although different breast carcinoma subtypes demonstrate specific MRI characteristics, heterogeneity in tumor presentation is observed even within the same subtype. Tumor imaging features on MRI reflect its epigenetic phenotype, modulated by genetic determinants as well as other biological and environmental factors. Taken together, these findings suggest that the biological behavior of breast carcinomas may be influenced by multiple factors beyond genetic constitution, with implications for treatment response and prognosis. This study aimed to evaluate the association between MRI tumor phenotype within different molecular subtypes and disease-free survival.

## 2. Materials and Methods

### 2.1. Methodology

This is a retrospective, single-center study approved by the Institutional Review Board (IRB) that evaluated 700 patients diagnosed with invasive breast carcinoma who underwent pre-treatment breast magnetic resonance imaging between October 2015 and May 2021.

**Figure 1:**
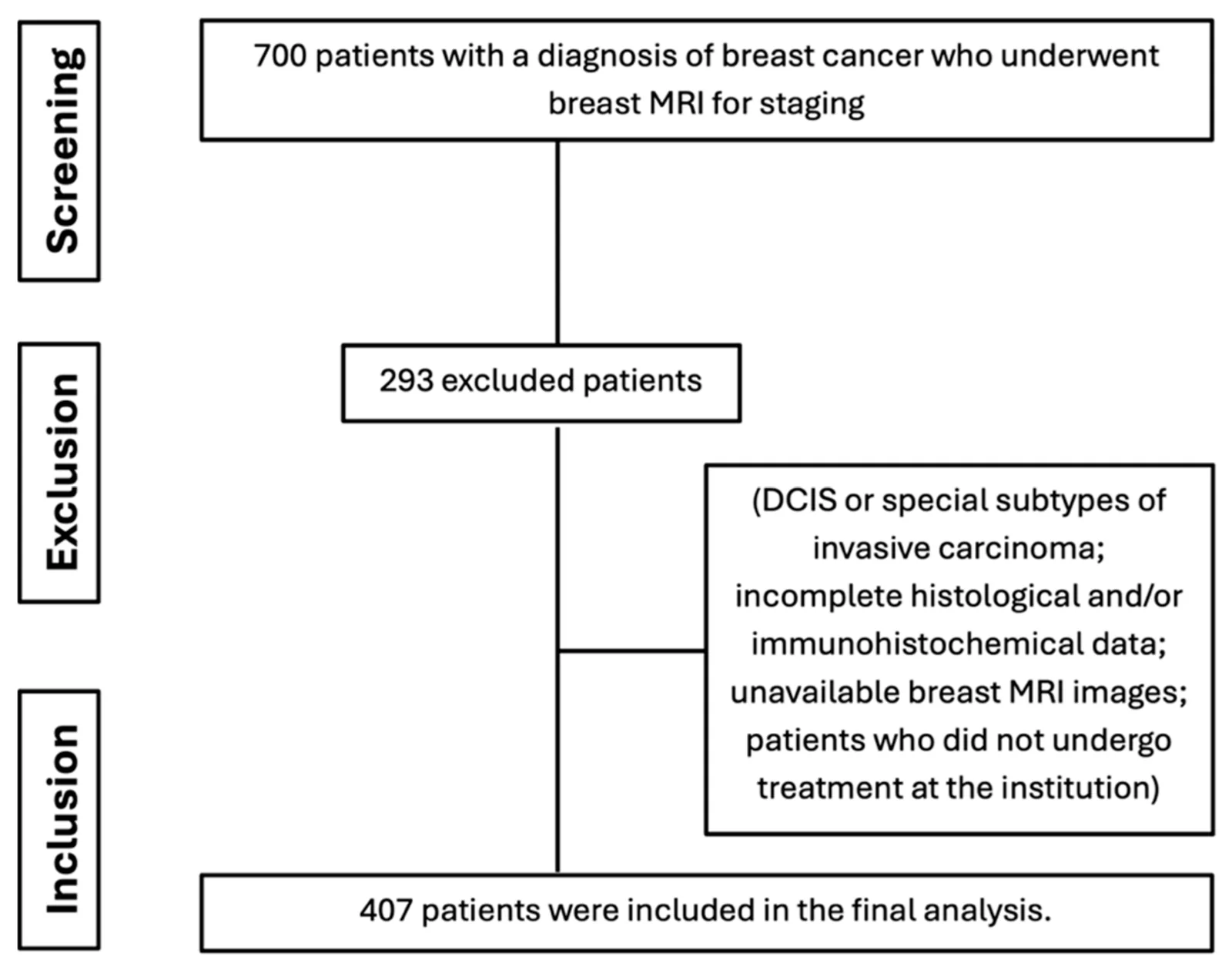
Flowchart of patient selection. DCIS, ductal carcinoma in situ; MRI, magnetic resonance imaging.

Patients with a diagnosis of ductal carcinoma in situ (DCIS) or special types of invasive breast carcinoma were excluded, as well as those lacking complete histological or immunohistochemical information, those whose MRI images were unavailable for analysis, and those who were not treated at the institution, totaling 293 excluded patients. Because exclusions were mainly due to incomplete histopathological, imaging, or follow-up information, a formal comparison between included and excluded patients was not possible, and selection bias cannot be excluded. Consequently, 407 patients with invasive carcinoma of no special type (NST) were included in the study (Figure 1).

Clinical follow-up data on recurrence, distant metastasis, and survival status were obtained by reviewing the institutional electronic medical records and oncology follow-up documentation. Follow-up time was calculated from the date of breast cancer diagnosis to the date of recurrence, distant metastasis, or last clinical follow-up. Patients without events were censored at the date of the last available follow-up. Patients lost to follow-up were censored at the date of their last available clinical information. Follow-up data were updated until database closure in February 2025. The median follow-up, estimated using the reverse Kaplan–Meier method, was 57 months.

### 2.2. Histological and Immunohistochemical Analysis

Immunohistochemical analysis of specimens obtained from pre-treatment biopsies was performed in accordance with the recommendations of the American College of Pathology. Estrogen receptor (ER) expression was considered positive when 1% or more of tumor cells showed nuclear staining of any intensity, and tumors were classified as ER-low positive when ER staining was present in 1–10% of tumor cells.

HER2 expression was considered positive when there was HER2 protein overexpression (IHC score 3+) or an IHC score of 2+ with HER2 gene amplification detected by fluorescence in situ hybridization (FISH) or dual in situ hybridization (DISH).

Immunohistochemical data were used to classify breast carcinomas into five IHC-defined (surrogate) immunophenotypes:

Luminal A (ER positive > 10%, HER2 negative, Ki-67 < 15%, and PgR ≥ 20%).

Luminal B (ER positive > 10%, HER2 negative, and Ki-67 ≥ 15% or PgR < 20%).

Luminal ER-low positive (ER positive 1–10% and HER2 negative).

HER2 (HER2 overexpression, ER negative or ER positive).

Triple negative (ER negative and HER2 negative).

For survival analyses, ER-low tumors (*n* = 26) were grouped with triple-negative tumors (*n* = 76) due to their similar biological behavior and clinical outcomes reported in the literature.

### 2.3. Treatment Protocols

During the study period, patients were treated according to standardized institutional protocols based on disease stage and molecular subtype. Adjuvant radiotherapy was routinely administered after breast-conserving surgery, and patients with luminal tumors received adjuvant endocrine therapy. Indications for neoadjuvant chemotherapy included HER2 and triple-negative/ER-low tumors larger than 2 cm, as well as T3/T4 or node-positive luminal tumors. Standard neoadjuvant regimens included AC-T (doxorubicin plus cyclophosphamide followed by paclitaxel) for luminal tumors; AC-THP (doxorubicin plus cyclophosphamide followed by paclitaxel, trastuzumab, and pertuzumab), THP (docetaxel, trastuzumab, and pertuzumab), or TCHP (docetaxel, carboplatin, trastuzumab, and pertuzumab) for HER2-positive tumors; and AC-TC (doxorubicin plus cyclophosphamide followed by paclitaxel and carboplatin) for triple-negative/ER-low tumors. Therefore, at our institution, ER-low tumors received the same systemic treatment as triple-negative tumors.

### 2.4. MRI Analysis

Magnetic resonance imaging examinations were performed on a high-field system (1.5 Tesla, Achieva, Philips Healthcare, Best, The Netherlands) using a dedicated 8-channel breast coil. Images were acquired before and after administration of the paramagnetic contrast agent gadopentetate dimeglumine (Gd-DTPA; dose of 20 mL; infusion rate of 3 mL/s). Prior to Gd-DTPA administration, a T1-weighted gradient-echo three-dimensional (3D) sequence was acquired in the axial plane (TR/TE, 627/8.0 ms; slice thickness, 3 mm; matrix, 280 × 399; field of view [FOV], 250 mm). A fat-suppressed short tau inversion recovery (STIR) sequence in the sagittal plane of both breasts was also acquired (TR/TE, 5127/80 ms; slice thickness, 3 mm; matrix, 220 × 208; FOV, 220 mm).

Five T1-weighted gradient-echo 3D axial phases were obtained using fat suppression for dynamic contrast-enhanced imaging (TR/TE, 5.1/2.5 ms; slice thickness, 1 mm; matrix, 352 × 429; FOV, 300 mm). The first phase was acquired before contrast injection, the second phase was performed 20 s after contrast injection, and subsequent phases were acquired in the following minutes with a temporal resolution of 60–90 s. Pre-contrast images were subtracted from post-contrast images to improve visualization of enhancing areas. The final sequence consisted of a sagittal 3D T1-weighted gradient-echo sequence with fat suppression (TR/TE, 5.5/2.9 ms; slice thickness, 1 mm; matrix, 368 × 364; FOV, 220 mm).

Pre-treatment MRI examinations were independently reviewed by two radiologists with 4 and 10 years of experience in breast imaging, who were blinded to the histopathological results, to assess tumor imaging features according to the Breast Imaging Reporting and Data System (BI-RADS^®^) lexicon. Evaluated features included lesion type (mass and non-mass enhancement [NME]) and lesion size. For mass lesions, shape (oval, round, or irregular), margins (circumscribed, irregular, or spiculated), internal enhancement (homogeneous, heterogeneous, or rim enhancement), and kinetic curve characteristics—initial phase (slow, medium, or fast) and delayed phase (persistent, plateau, or washout)—were assessed. For NME lesions, distribution patterns (focal, linear, segmental, regional, multiple regions, or diffuse) were evaluated. Imaging phenotype classification was performed independently by the two radiologists, and discrepancies were resolved by consensus.

### 2.5. Tumor Classification

**Figure 2:**
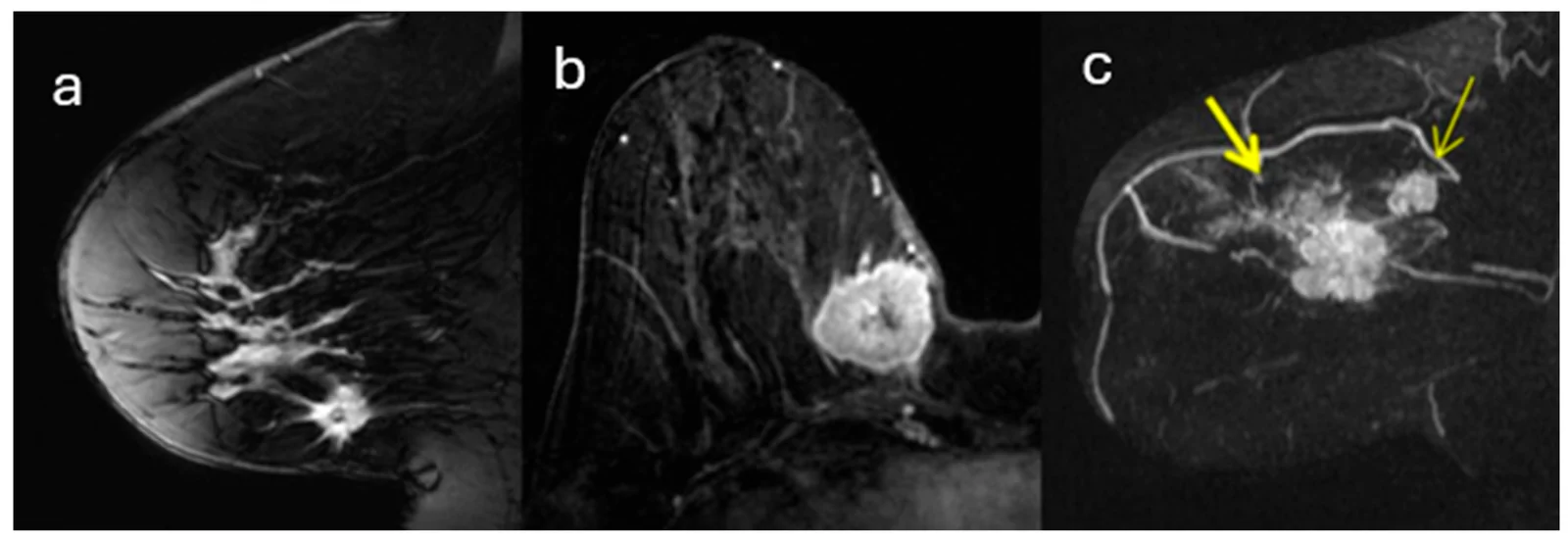
MRI phenotypes and their association with molecular subtypes. Sagittal (**a**,**c**) and axial (**b**) contrast-enhanced T1-weighted fat-saturated breast MRI sequences. (**a**) Type 1 is characterized by a unifocal mass with an irregular shape and spiculated margins. (**b**) Type II is characterized by a unifocal mass with an oval or round shape and circumscribed margins, which may exhibit a peripheral (rim) enhancement pattern with central necrosis. (**c**) Type III includes lesions that do not meet the criteria for Type 1 or 2, as well as the presence of non-mass enhancement areas (thick arrow) or multifocal/multicentric disease (thin arrow).

Based on breast MRI characteristics, tumors were classified into three imaging phenotypes, according to a previously published framework correlating imaging features with different tumor subtypes [16], and are shown in Figure 2:

Type 1: Characterized by a unifocal mass with irregular shape and spiculated margins, this phenotype is most observed in Luminal A subtype tumors.

Type 2: Characterized by a unifocal mass with oval or round shape and circumscribed margins, which may exhibit a peripheral (rim) enhancement pattern with central necrosis, this phenotype is most observed in triple-negative (TN) and ER-low tumors.

Type 3: Comprising lesions that do not meet the above criteria, as well as the presence of non-mass enhancement areas or multifocal/multicentric disease, this phenotype is most observed in Luminal B or HER2 subtype tumors.

### 2.6. Statistical Analysis

Initially, a descriptive analysis of the variables was performed, with absolute (*n*) and relative (%) frequency distributions presented. The relationship between different molecular subtypes and imaging appearance was assessed using cross-tabulation, and the association between them was evaluated using the chi-square test of independence.

For disease-free survival analysis, evaluations were conducted separately for each of the four subtypes: Luminal A, Luminal B, HER2 overexpressed, and TN/ER low. Survival times were calculated as the interval between the date of diagnosis and the date of recurrence/distant metastasis or the last follow-up. Death without previously documented recurrence was not considered a DFS event, and these patients were censored at the date of death. Only three patients died without previously documented recurrence (two in the Luminal B and one in the HER2-overexpressed subgroup; none in the triple-negative/ER-low subgroup), so the analyses in the triple-negative/ER-low subgroup were unaffected by this censoring.

Patients who did not experience an event by the previous follow-up date were considered censored. Kaplan–Meier survival curves were constructed, and differences between curves for variables of interest were assessed using the log-rank test. Mean survival times were estimated as restricted mean survival times, defined as the area under the Kaplan–Meier curve at the longest observed follow-up time within each subtype, with the same time horizon applied across comparison groups within each subtype.

Subsequently, assumptions and frequency distributions were evaluated to construct a Cox proportional hazards model to estimate the hazard ratio (HR) and better quantify and understand the observed differences [1]. In addition to molecular subtype, the following variables of interest were included in the models: age, T stage, N stage, imaging phenotype, and pathological response (in patients who underwent neoadjuvant chemotherapy). Pathological response (residual cancer burden, RCB) was available only for patients treated with neoadjuvant chemotherapy; analyses including this variable were therefore restricted to this subgroup. Given the limited number of outcome events and the small number of patients in some exposure categories, a sensitivity analysis was performed using Firth-penalized Cox proportional hazards regression to reduce potential small-sample and sparse-data bias. The penalized model included the same covariates as the primary multivariable Cox model, and 95% confidence intervals and *p*-values were obtained using profile penalized likelihood.

A significance level of 5% was adopted for all statistical tests in the study, and statistical analyses were performed using the R software environment (version 3.6.2).

## 3. Results

The study population comprised 407 patients, with a mean age of 50 years (range: 18–86 years). Of these, 50 patients (12.28%) were of the Luminal A subtype, 191 (46.92%) Luminal B, 64 (15.72%) HER2 overexpressed, and 102 (25.06%) triple negative/ER low. Table 1 summarizes clinical data, MRI findings, histological features, and follow-up outcomes by tumor subtype. The median follow-up time was 57 months (95% CI: 55.08–58.91).

When comparing the four subgroups, the Luminal A subtype demonstrated the most indolent profile, with a higher proportion of older patients (60% ≥ 50 years), smaller tumors (58% < 20 mm), lower stage, lower nuclear and histological grade, and more-favorable outcomes (mortality 4%, metastasis 4%). Luminal B tumors also predominantly occurred in older patients (55.5% ≥ 50 years); however, they were associated with larger tumors, higher histological and nuclear grades, and worse outcomes (mortality 5.8%; metastasis 12.6%). The HER2-overexpressed subtype included a higher proportion of younger patients (64.1% < 50 years) and larger tumors (82.8% ≥ 20 mm), with greater lymph node involvement on MRI, high histological and nuclear grades, and intermediate outcomes (mortality 4.7%, metastasis 9.4%). Finally, the triple-negative/ER-low subtype exhibited the most aggressive profile, characterized by larger tumors, more frequent axillary involvement, high histological and nuclear grades, and the poorest outcomes (mortality 16.7%, metastasis 22.5%).

Regarding imaging phenotype, Type 3 was the most common (60.0%), followed by Type 2 (21.9%) and Type 1 (74/407; 18.2%). Imaging phenotype Type 1 was most frequently observed in the Luminal A subtype. In contrast, Type 2 was more common in the triple-negative/ER-low subtype, and Type 3 predominated in the Luminal B and HER2-overexpressed subtypes (Table 2).

The Kaplan–Meier estimated recurrence-free survival was approximately 78% at the end of the follow-up period. Table 3 presents the estimated restricted mean survival times and the number of events in each category. Table 4 shows the results of the univariable Cox models for the Luminal B, HER2-overexpressed, and triple-negative/ER-low subtypes. It was not possible to construct a model for the Luminal A subtype due to the small number of events in some subgroups.

T stage was associated with disease-free survival (DFS) in the Luminal B and triple-negative/ER-low groups. For the Luminal B subtype, patients with T3/T4 stage tumors had a 2.34-fold higher risk of recurrence compared with those with T1/T2 tumors. For the HER2-overexpressed subtype, the T3/T4 group had a 5.13-fold higher risk of recurrence compared with the T1/T2 group, although this association did not reach statistical significance (*p* = 0.062). For the triple-negative/ER-low subtype, the T3/T4 group had a 3.72-fold higher risk of recurrence compared with the T1/T2 group. In addition to T stage, in the triple-negative/ER-low subgroup, imaging phenotype was associated with DFS in the univariable Cox model, and pathological response to neoadjuvant chemotherapy (NAC) showed a borderline association (*p* = 0.051): patients with partial response to NAC (RCB I–III) had a 2.82-fold higher risk of recurrence compared with those with complete response (RCB 0); patients with imaging phenotype Type 1 had a 10.01-fold higher risk, and those with imaging phenotype Type 3 had a 2.80-fold higher risk of recurrence compared with those with Type 2 phenotype.

Table 5 presents the multivariable Cox model for the triple-negative/ER-low subtype, which included the covariates T stage, pathological response, and imaging phenotype, selected based on clinical relevance and the results of the univariable analyses (pathological response showed borderline significance, *p* = 0.051); age and N stage were not included because they showed no association with DFS in the univariable analysis (*p* = 0.264 and *p* = 0.977, respectively). Because pathological response was available only for patients treated with NAC, the multivariable model comprised 80 patients with 17 DFS events. Only the imaging phenotype remained significant in the multivariable model, indicating that patients with imaging phenotype Type 1 had an 8.16-fold higher risk of recurrence compared with those with Type 2 (95% CI: 1.49–44.60; *p* = 0.015). In a sensitivity analysis using Firth-penalized Cox regression, the association between Type 1 imaging pattern and the outcome remained similar to that observed in the conventional multivariable Cox model (HR of 7.80; 95% CI 1.53–40.55; *p* = 0.015). This estimate was based on 5 of the 8 patients with the Type 1 phenotype (3 of whom experienced events), 35 of the 45 patients with the Type 2 phenotype (3 with events), and 40 of the 49 patients with the Type 3 phenotype (11 with events); the wide confidence interval reflects substantial instability, and this result should be interpreted with caution. Moreover, because RCB is a post-treatment variable available only for patients who received NAC, the adjusted estimate reflects the association between imaging phenotype and DFS conditional on pathological response within the neoadjuvant subgroup, rather than the independent prognostic value of the baseline MRI phenotype in the entire population.

**Figure 3:**
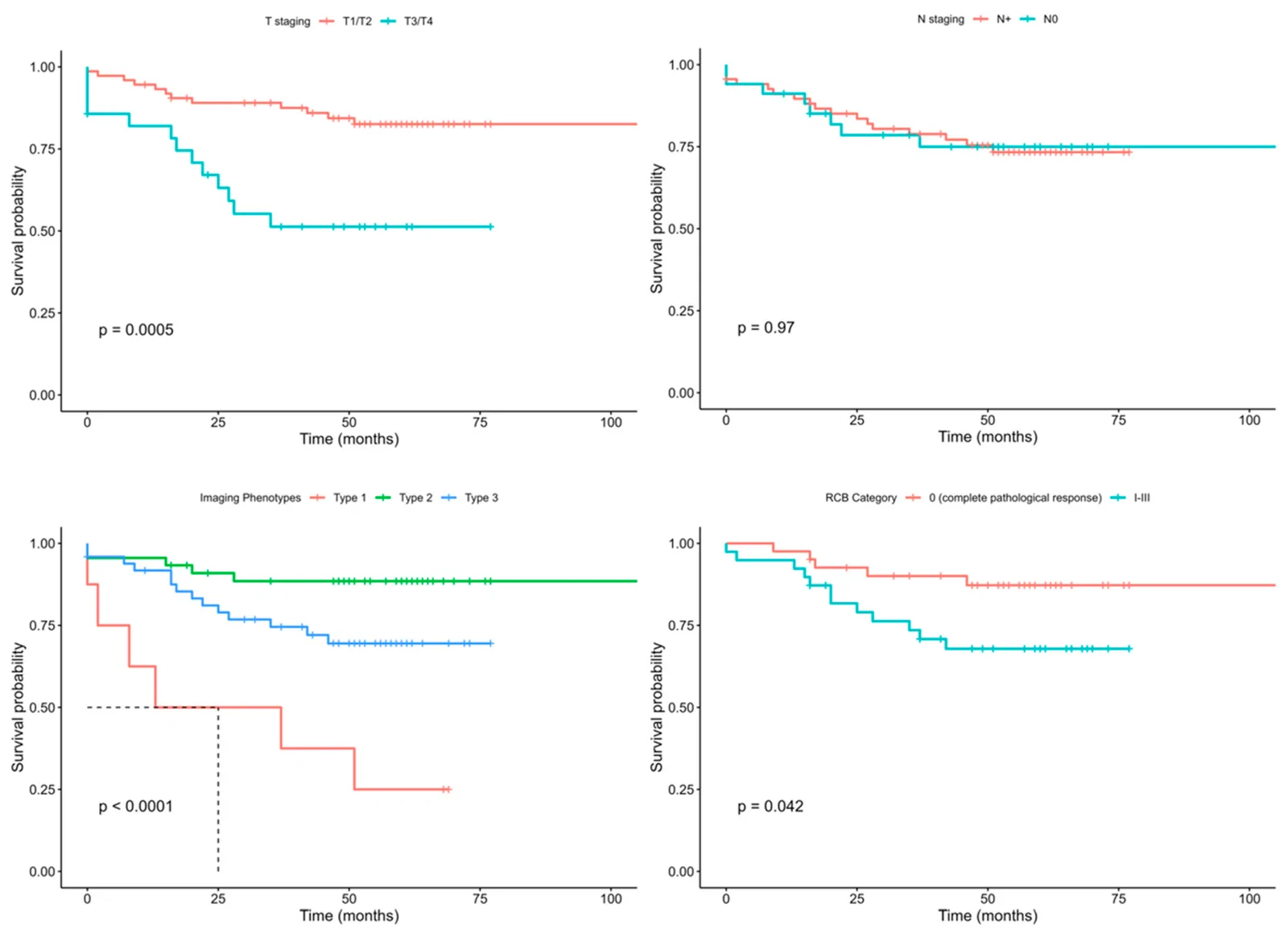
Kaplan–Meier Disease-Free Survival (DFS) Curves for Triple-Negative/ER-low breast cancer subtype.

Figure 3 shows the survival curves by variables of interest for the triple-negative/ER-low subtype, demonstrating significant differences among curves for T stage (*p* < 0.001), imaging phenotype (*p*-value < 0.001), and RCB (*p*-value = 0.042). The groups with the highest survival were those with T1/T2 stage tumors, imaging phenotype Type 2, and RCB 0.

## 4. Discussion

In this study, we evaluated the association between imaging phenotype and survival in patients with different breast cancer subtypes. To this end, breast carcinoma imaging features on MRI were categorized into three groups (Types 1, 2, and 3) based on morphology, margins, and distribution patterns. No significant association was observed between imaging phenotype and prognosis in the Luminal A, Luminal B, or HER2-overexpressed subtypes. However, in tumors of the TN/ER-low subtype, imaging phenotype was statistically associated with disease-free survival.

Overall, TN and ER-low tumors more frequently exhibited Type 2 lesions, characterized by unifocal, circumscribed, or round masses with peripheral enhancement. This pattern has previously been described as typical of TN tumors, which often present as well-defined masses on MRI despite their biologically aggressive behavior [6,12]. Our findings suggest that, within this heterogeneous group, a more circumscribed morphology may be associated with a better response to systemic therapy and, consequently, improved survival, supporting studies that have identified TN subgroups with a less unfavorable prognosis [6].

Müller et al. also highlighted this phenomenon, describing substantial heterogeneity in imaging features in TN tumors and emphasizing less aggressive patterns that may reflect meaningful biological differences [17].

Conversely, we observed that cases classified as Type 1 (unifocal tumors with spiculated margins) and Type 3 (multifocal or multicentric lesions with a non-mass enhancement component) were associated with worse survival outcomes. Although such patterns are more commonly described in HER2-positive and high-grade luminal tumors [5,18], some TN tumors in our cohort also exhibited spiculated margins—an imaging finding less commonly observed in this subtype. In most cases, TN tumors present as a unifocal enhancing mass on MRI [19]. Thus, even within the TN group, morphological heterogeneity on MRI may provide valuable prognostic clues.

Another relevant aspect is the potential role of the androgen receptor (AR). Recent evidence indicates that AR expression may modulate tumor behavior in a subset of TN carcinomas, being associated with slower growth patterns and imaging features more like luminal tumors [20,21]. Studies such as that by Dogra et al. have shown that AR expression in a significant fraction of TN tumors may represent not only a prognostic marker but also a potential therapeutic target [22]. Because AR expression was not assessed in the present study, any relationship between imaging phenotype and AR status remains speculative and is proposed here only as a hypothesis to be tested in future studies.

Kaplan–Meier survival analysis demonstrated significant differences among imaging phenotypes, although the limited sample size in some subgroups warrants cautious interpretation. Previous studies have already indicated that multifocality is associated with a higher risk of recurrence and worse prognosis [23,24]. Because the Type 3 category is intrinsically heterogeneous, encompassing multifocal/multicentric disease and non-mass enhancement, the poorer outcomes observed in this group may be partly driven by multifocality itself rather than by lesion morphology alone.

Overall, circumscribed, round, and unifocal lesions (Type 2) were associated with better survival. In contrast, multifocal or multicentric presentations, or those with spiculated margins (Types 1 and 3), were associated with worse outcomes. These findings suggest that MRI morphology, even within the TN group, may provide prognostic information, although confirmation in larger cohorts is required before any therapeutic implications can be drawn.

This study has limitations related to its retrospective, single-center design and the relatively small sample size. In addition, it was not possible to include analyses from other imaging modalities, such as mammography and ultrasonography. Additional limitations should be acknowledged. The main finding in the triple-negative/ER-low group was based on a very small Type 1 subgroup (8 patients, 6 events), resulting in a wide confidence interval and an unstable risk estimate. Although a sensitivity analysis using Firth-penalized Cox regression yielded consistent results, the hazard ratios in this subgroup remain imprecise and should be regarded as exploratory. The multivariable model included several covariates relative to the limited number of events, raising the possibility of overfitting; its estimates should therefore be regarded as exploratory. Age and N stage could not be added to the model without further compromising the events-per-variable ratio, and residual confounding by these variables cannot be excluded. Pathological response data were available only for patients treated with neoadjuvant chemotherapy, and detailed treatment information (systemic therapy, surgery, and radiotherapy) was not analyzed. Detailed information on systemic therapy, surgery, radiotherapy, endocrine therapy, and HER2-targeted therapy was unavailable; although subtype-stratified analyses and the use of standardized institutional treatment protocols may have reduced treatment-related heterogeneity, residual confounding affecting DFS cannot be excluded. Analyses were performed separately within each subtype, and no formal interaction test between imaging phenotype and molecular subtype was conducted; thus, differences in prognostic value among subtypes cannot be formally established. Although imaging phenotype was assessed independently by two radiologists with discrepancies resolved by consensus, formal inter-reader agreement was not measured. The grouping of triple-negative and ER-low tumors, although biologically plausible, combines entities that are not identical from a clinical or therapeutic standpoint. Finally, the exclusion of a substantial proportion of screened patients (41.9%) may have introduced selection bias; no correction for multiple comparisons was applied. Nevertheless, the standardized imaging classification and direct comparison across molecular subtypes add originality and relevance to the results.

## 5. Conclusions

This exploratory analysis suggests that MRI phenotype may provide additional prognostic information in TN and ER-low tumors. Circumscribed, round, and unifocal lesions were associated with better survival, whereas multifocal, multicentric, or spiculated-margin presentations were associated with worse outcomes. These exploratory findings do not yet support therapeutic individualization; larger studies are needed before MRI morphology can be applied for risk stratification in these subtypes. Furthermore, the potential relationship between imaging phenotype and androgen receptor expression, which this study did not assess, remains a hypothesis for future investigation. Our findings underscore the relevance of MRI not only for detection and staging but also as a potential complementary tool for risk stratification in subtypes with an unfavorable prognosis, pending external validation.

## Figures and Tables

**Table 1:** Characteristics of the included patients, according to breast cancer subtype.

Variable	Category	Luminal A	Luminal B	HER2 Overexpressed	Triple Negative/ER Low	Total
Age	<50	20 (40%)	85 (44.5%)	41 (64.1%)	72 (70.6%)	218 (53.6%)
	≥50	30 (60%)	106 (55.5%)	23 (35.9%)	30 (29.4%)	189 (46.4%)
Tumor size (main lesion)	<20 mm	29 (58%)	68 (35.6%)	11 (17.2%)	30 (29.4%)	138 (33.9%)
	≥20 mm	19 (38%)	120 (62.8%)	53 (82.8%)	72 (70.6%)	264 (64.9%)
	NA	2 (4%)	3 (1.6%)	0 (0.0%)	0 (0.0%)	5 (1.2%)
Tumor distribution	Multifocal	13 (26%)	51 (26.7%)	18 (28.1%)	17 (16.7%)	99 (24.3%)
	Unifocal	37 (74%)	140 (73.3%)	46 (71.9%)	85 (83.3%)	308 (75.7%)
Lesion type	NME	8 (16%)	27 (14.1%)	12 (18.8%)	7 (6.9%)	54 (13.3%)
	Mass	40 (80%)	137 (71.7%)	42 (65.6%)	84 (82.4%)	303 (74.4%)
	Mass + NME	2 (4%)	27 (14.1%)	10 (15.6%)	11 (10.8%)	50 (12.3%)
T stage (MRI)	T1/T2	41 (82%)	145 (75.9%)	41 (64.1%)	74 (72.5%)	301 (74%)
	T3/T4	7 (14%)	44 (23%)	23 (35.9%)	28 (27.5%)	102 (25.1%)
	NA	2 (4%)	2 (1%)	0 (0.0%)	0 (0.0%)	4 (1%)
N stage (MRI)	N+	15 (30%)	74 (38.7%)	43 (67.2%)	68 (66.7%)	200 (49.1%)
	N0	35 (70%)	116 (60.7%)	21 (32.8%)	34 (33.3%)	206 (50.6%)
	NA	0 (0.0%)	1 (0.5%)	0 (0.0%)	0 (0.0%)	1 (0.2%)
Histological grade	I	28 (56%)	30 (15.7%)	3 (4.7%)	1 (1%)	62 (15.2%)
	II	19 (38%)	105 (55%)	23 (35.9%)	19 (18.6%)	166 (40.8%)
	III	1 (2%)	55 (28.8%)	35 (54.7%)	76 (74.5%)	167 (41%)
	NA	2 (4%)	1 (0.5%)	3 (4.7%)	6 (5.9%)	12 (2.9%)
Nuclear grade	1	12 (24%)	5 (2.6%)	1 (1.6%)	0 (0.0%)	18 (4.4%)
	2	30 (60%)	87 (45.5%)	6 (9.4%)	10 (9.8%)	133 (32.7%)
	3	8 (16%)	98 (51.3%)	57 (89.1%)	92 (90.2%)	255 (62.7%)
	NA	0 (0.0%)	1 (0.5%)	0 (0.0%)	0 (0.0%)	1 (0.2%)
RCB	0	0 (0.0%)	9 (4.7%)	30 (46.9%)	41 (40.2%)	80 (19.7%)
	I–III	1 (2%)	44 (23%)	20 (31.2%)	39 (38.2%)	104 (25.6%)
	NA	49 (98%)	138 (72.3%)	14 (21.9%)	22 (21.6%)	223 (54.8%)
Status	Alive	48 (96%)	178 (93.2%)	61 (95.3%)	84 (82.4%)	371 (91.2%)
	Death	2 (4%)	11 (5.8%)	3 (4.7%)	17 (16.7%)	33 (8.1%)
	NA	0 (0.0%)	2 (1%)	0 (0.0%)	1 (1%)	3 (0.7%)
Local recurrence	No	50 (100%)	180 (94.2%)	64 (100%)	97 (95.1%)	391 (96.1%)
	Yes	0 (0.0%)	9 (4.7%)	0 (0.0%)	5 (4.9%)	14 (3.4%)
	NA	0 (0.0%)	2 (1%)	0 (0.0%)	0 (0.0%)	2 (0.5%)
Distant metastasis	No	48 (96%)	166 (86.9%)	58 (90.6%)	79 (77.5%)	351 (86.2%)
	Yes	2 (4%)	24 (12.6%)	6 (9.4%)	23 (22.5%)	55 (13.5%)
	NA	0 (0.0%)	1 (0.5%)	0 (0.0%)	0 (0.0%)	1 (0.2%)

MRI, magnetic resonance imaging; HER2, human epidermal growth factor receptor 2; NME, non-mass enhancement; T stage, primary tumor stage; N stage, regional lymph node stage; Scarff–Bloom–Richardson grading system; RCB, residual cancer burden; NA, not available; ER, estrogen receptor; progesterone receptor.

**Table 2:** Association between breast cancer subtype and imaging phenotype on MRI.

		Luminal A	Luminal B	HER2 Overexpressed	Triple Negative/ER Low	Total	*p*-Value
Imaging Phenotype	Type 1	15 (30%)	41 (21.5%)	10 (15.6%)	8 (7.8%)	74 (18.2%)	<0.0001
	Type 2	6 (12%)	31 (16.2%)	7 (10.9%)	45 (44.1%)	89 (21.9%)	
	Type 3	29 (58%)	119 (62.3%)	47 (73.4%)	49 (48%)	244 (60%)	
	Total	50 (100%)	191 (100%)	64 (100%)	102 (100%)	407 (100%)	

HER2, human epidermal growth factor receptor 2; ER, estrogen receptor.

**Table 3:** Number of events and restricted mean survival time for Disease-Free Survival (DFS) analysis in breast cancer patients according to subtype, clinicopathological and imaging features.

Subtype	Variable	Category	N	Number of Events	Mean Survival Time
Luminal B	Age	<50	85	13	88.8
		≥50	105	17	87.4
	T stage (MRI)	T1/T2	144	18	90.41
		T3/T4	44	12	78.72
	N stage (MRI)	N+	74	16	83.97
		N0	115	14	90.06
	RCB	0	9	0	102
		I–III	44	6	90.99
	Imaging phenotype	Type 1	41	4	93.84
		Type 2	31	5	87.12
		Type 3	118	21	85.71
HER2 overexpressed	Age	<50	41	3	73.15
		≥50	23	3	70.85
	T stage (MRI)	T1/T2	41	2	76.44
		T3/T4	23	4	67.1
	N stage (MRI)	N+	43	5	71.75
		N0	21	1	73.5
	RCB	0	30	0	79
		I–III	20	2	72.2
	Imaging phenotype	Type 1	10	2	72.67
		Type 2	7	0	79
		Type 3	47	4	73.09
Triple negative/ER low	Age	<50	72	15	87.72
		≥50	30	10	78.39
	T stage (MRI)	T1/T2	74	12	93.17
		T3/T4	28	13	63.03
	N stage (MRI)	N+	68	17	84.85
		N0	34	8	84.82
	RCB	0	41	5	97.23
		I–III	39	12	80.23
	Imaging phenotype	Type 1	8	6	40.88
		Type 2	45	5	97.06
		Type 3	49	14	81.46

MRI, magnetic resonance imaging; HER2, human epidermal growth factor receptor 2; ER, estrogen receptor; RCB, residual cancer burden; N, number of patients.

**Table 4:** Univariable Cox Regression Models to predict disease-free survival (DFS) according to breast cancer subtypes.

Subtype	Variable	Category	HR	CI (95%)		*p*-Value	Schoenfeld Test
Luminal B	Age	≥50	1.1615	0.5628	2.3967	0.6855	0.0894
	T stage	T3/T4	2.3464	1.1287	4.8779	0.0224	0.8240
	N stage	N+	1.7314	0.8449	3.5482	0.1337	0.9874
	Imaging phenotype	Type 1	0.5747	0.1542	2.1420	0.4092	0.9795
		Type 3	1.0785	0.4065	2.8615	0.8794	
HER2 overexpressed	Age	≥50	1.4022	0.2744	7.1654	0.6847	0.0338
	T stage	T3/T4	5.1326	0.9230	28.5410	0.0617	0.0728
	N stage	N+	1.9801	0.2249	17.4312	0.5382	0.3918
Triple negative/ER low	Age	≥50	1.5780	0.7089	3.5128	0.2639	0.1336
	T stage	T3/T4	3.7225	1.6915	8.1921	0.0011	0.7437
	N stage	N+	1.0124	0.4368	2.3464	0.9772	0.4708
	RCB	I–III	2.8232	0.9941	8.0182	0.0513	0.9598
	Imaging phenotype	Type 1	10.0187	3.0468	32.9443	0.0001	0.4914
		Type 3	2.8029	1.0089	7.7870	0.0480	

HR, hazard ratio; CI, confidence interval; HER2, human epidermal growth factor receptor 2; ER, estrogen receptor; RCB, residual cancer burden; T stage, primary tumor stage; N stage, regional lymph node stage.

**Table 5:** Multivariable Cox Proportional Hazards Model to predict disease-free survival (DFS) for Triple-Negative/ER-low subtype.

Variable	Category	HR	CI (95%)		*p*-Value	Schoenfeld Test
T stage	T3/T4	2.0394	0.6757	6.1548	0.2060	
RCB	I–III	2.0322	0.6650	6.2102	0.2134	0.7683
Imaging phenotype	Type 1	8.1592	1.4928	44.5956	0.0154	
	Type 3	2.7581	0.7413	10.2613	0.1301	

HR, hazard ratio; CI, confidence interval; RCB, residual cancer burden; T stage, primary tumor stage.

## Data Availability

The data presented in this study are available on request from the corresponding author. The data are not publicly available due to privacy/ethical restrictions.
